# C-751-07. Self-esteem, Guilt, and Shame in Adult Burn Survivors: Is There a Connection?

**DOI:** 10.1093/jbcr/irag033.012

**Published:** 2026-04-06

**Authors:** Daniel W Chacon, Ruth B Rimmer, C L C P PhD, Cindy Rutter

**Affiliations:** Phoenix Society for Burn Survivors, Fresno, CA; Care Plans For Life, Phoenix, AZ; 1 Amazing Life, Carlsbad, CA

## Abstract

**Introduction:**

Self-esteem and self-conscious emotions (guilt, shame) may shape psychosocial recovery after burn injury. This study sought to determine if an association between self-esteem and guilt/shame proneness exists in burn-injured adults with gender-stratified analyses and exploratory checks across demographic and burn-related factors.

**Methods:**

Participants (n = 40) at a burn retreat voluntarily completed a post-retreat survey. Measures included the Rosenberg Self-Esteem Scale (0–30) and the Guilt and Shame Proneness Scale (four 1–7 subscales: Guilt-NBE, Guilt-Repair, Shame-NSE, Shame-Withdrawal; composites and total mean). Demographics collected: age, gender, years since burn, TBSA (banded), burn mechanism, scar visibility (hidden/visible/both), and perceived family support. Primary analyses were Pearson correlations between self-esteem and GASP metrics and one-way ANOVA of self-esteem by gender; exploratory tests examined scar visibility and family support.

**Results:**

Participants included burn survivors (n = 40), with a mean age of 38.35 years, SD (±15.64), female (n = 26), male (n = 12), Nonbinary (n = 2) Caucasian (37.5%), Hispanic (37.5%), and African American (12.5%), the average age at burn was 17 years, with 77% reporting visible burn scars. Mean RSES was 17.7 (SD 2.85). RSES correlated negatively with the Guilt composite (r = −0.40, *p*=.011), Guilt-Repair (r = −0.37, *p*=.019), Shame-NSE (r = −0.34, *p*=.034), and GASP total (r = −0.37, *p*=.018); RSES differed by gender (F = 6.65, *p*=.003, η^2^ = .26). No significant differences in RSES by scar visibility (*p*=.16) or family support.

**Conclusions:**

Among adult burn survivors, lower self-esteem aligned with higher guilt proneness; especially repair-oriented guilt and with shame-based negative self-evaluation. Because shame is a non-productive emotion often leading to negative and detrimental behaviors, this finding is of concern. As in other studies, female survivors are experiencing lower self-esteem. Findings point toward targeting guilt and shame levels of survivors while with consideration for gender-specific patterns in psychosocial support.

**Applicability of Research to Practice:**

Burn support programs may consider a brief screening for guilt/shame and providing gender-focused interventions (e.g., cognitive restructuring, self-compassion work, repair-focused skills). Given the null effects for scar visibility and family support here, psychosocial targets may be more actionable than injury characteristics alone; larger samples should confirm subgroup effects and test change over time.

**Funding for the study:**

N/A.

